# Spatially resolved host–fungal transcriptomics profiling by fungal-DBiT

**DOI:** 10.64898/2026.10.19.760099

**Published:** 2026-10-19

**Authors:** Xiaohui Yu, Fu Gao, Deguang Song, Gaocai Li, Dejiang Wang, Shenjie Wang, Xiangjun Di, Shujie He, Liyun Lin, Junyi Xu, Di Zhang, Yu Jin, Jing Du, Noah W. Palm, Yang Liu

## Abstract

Hosts and their associated fungi coexist within a shared tissue environment, engaging in dynamic interactions that influence immune regulation, tissue homeostasis, and disease progression. Dissecting these interactions requires spatially resolved and unbiased approaches capable of capturing gene expression from both host and fungal cells within intact tissue architecture. Here, we present fungal-DBiT, a spatial transcriptomic platform that enables simultaneous, high-resolution mapping of host and fungal RNAs, directly in tissue sections. Using fungal-DBiT, we profiled the spatial distribution of host and fungal transcripts in a fungal infection mouse model, and review pronounced spatial heterogeneity in fungal localization and activity, accompanied by region-specific host transcriptional responses. We further applied fungal-DBiT to intestinal tissues under physiological conditions, demonstrating its sensitivity in detecting low-abundance fungal species in the absence of overt inflammation. Together, these results demonstrate the ability of fungal-DBiT to resolve host–fungal interactions in situ and highlight its utility for studying mycobiome-associated immune responses and disease processes with spatial precision.

